# Monkeypox in an Immunocompromised Patient: A Case Report

**DOI:** 10.7759/cureus.76645

**Published:** 2024-12-30

**Authors:** Andrea Mata Jimenez, Natalia Lopez

**Affiliations:** 1 Radiology Department, Universidad Internacional del Ecuador, Quito, ECU

**Keywords:** findings, imaging, immunocompromised, lungs, monkeypox

## Abstract

Monkeypox (Mpox) was declared a public health emergency in 2022. The most common form of presentation is a self-limited illness associated with a skin rash. However, immunocompromised patients may present with more severe forms of the disease. We present the case of an immunocompromised patient diagnosed with Mpox, who, in addition to presenting the characteristic skin lesions, also exhibited gastrointestinal involvement and incidental tomographic findings of pulmonary involvement due to the virus.

## Introduction

Monkeypox (Mpox) is a DNA virus of the *Orthopoxvirus* genus, belonging to the Poxviridae family, which also includes cowpox and other viruses. This virus can spread to humans and cause Mpox disease, which is quite similar to smallpox. There are two distinct clades of the virus: clade I and clade II [1,2].

In July 2022, the WHO declared Mpox a public health emergency due to a global outbreak that continues to affect some African countries. In addition, the incidence has been increasing in both endemic and non-endemic regions (such as the United Kingdom, the United States, Israel, and Singapore) [1,2].

Mpox is considered a zoonotic disease, but its natural reservoir remains unknown. However, several studies have suggested that small mammals, such as squirrels and monkeys, may be susceptible hosts [2,3].

Transmission of the virus occurs through animal-to-human and human-to-human contact. Recently, sexual transmission has been identified, particularly among men who have sex with other men [1].

Mpox typically causes a self-limited disease, with symptoms appearing up to 21 days after exposure and lasting from two to four weeks, depending on the patient’s immune system. The prodromal stage, lasting from zero to five days, is characterized by nonspecific symptoms such as headache, fever, lymphadenopathy, and myalgia. This is followed by the appearance of a skin rash that begins as itchy macules and papules, which progress to vesicles and pustules before forming scabs. The rash tends to spread centrifugally, with predominant involvement of the face and extremities, but it can also affect the oral, genital, rectal, conjunctival mucous membranes, and cornea [4,5].

There is evidence that immunosuppressed patients may develop complications, including generalized rashes, bacterial or fungal superinfection of skin lesions, intestinal obstruction, cardiac issues, and pulmonary, urinary, and neurological disorders, among others [1,6].

Patients are contagious from the onset of prodromal symptoms until the scabs have fallen off [4]. Diagnosis is confirmed by detecting viral DNA using polymerase chain reaction (PCR) from a sample taken directly from the lesion [2].

Treatment focuses on symptom control, and vaccination is recommended for immunosuppressed patients and those at higher risk of contracting the disease, particularly during an outbreak [2].

## Case presentation

A 57-year-old female patient with a history of rheumatoid arthritis and Sjögren's syndrome, who had been receiving methotrexate for six years, presented with diffuse abdominal pain of moderate intensity lasting for 24 hours, accompanied by nausea and diarrhea on one occasion, with no improvement in her condition.

**Figure 1:**
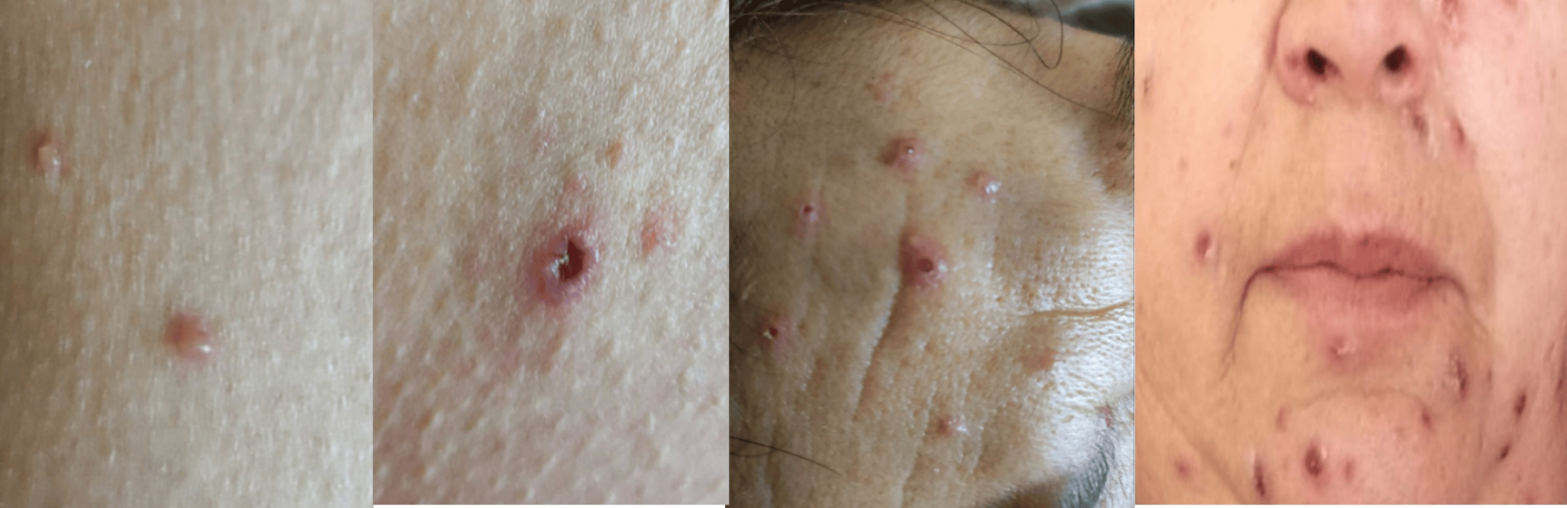
Papulopustular skin lesions distributed across the face.

On physical examination, the patient was stable, with vital signs within normal limits. The abdomen was soft and depressible but painful to deep palpation in the right iliac fossa. In addition, the patient reported a cough and headache and presented with several lesions spread across the skin in different stages of evolution, such as pustules, papules, vesicles, and scabs (Figure 1).

Therefore, the patient was questioned again and reported that she had traveled outside the city to a coastal area a month previously. Subsequently, she then decided to go to the hospital, where laboratory tests were performed due to suspicion of Mpox, given her travel history, which gave positive results.

**Figure 2:**
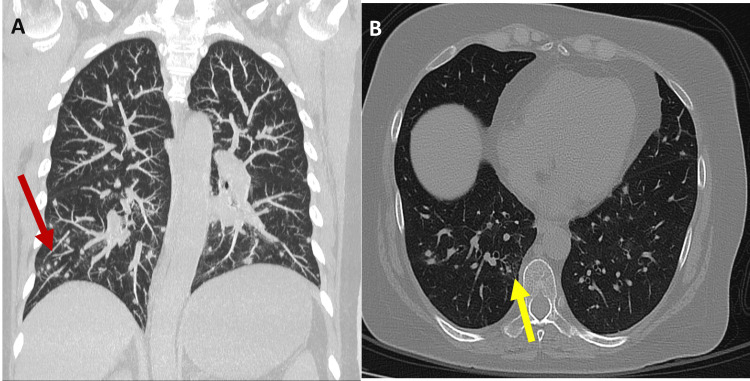
Chest CT in A) coronal plane MIP (maximum intensity projection) that shows several bilateral and diffuse solid nodules (red arrow), and B) axial plane MPR (multiplanar reformation) shows ground-glass pattern and atelectatic bands (yellow arrow).

Upon admission, the patient underwent a contrast-enhanced computed tomography (CT) scan of the chest, abdomen, and pelvis, which showed several solid nodules diffusely distributed in both lung fields, associated with a ground-glass pattern and atelectatic bands (Figure 2).

Laboratory tests showed only a C-reactive protein (CRP) of 6.5 mg/dl (reference value <0.3 mg/dl) with no other relevant findings. Therefore, the patient was admitted for symptomatic management of the abdominal pain with contact isolation due to her history.

Due to the persistence of abdominal pain, the patient underwent a colonoscopy, during which a sessile polyp with a regular surface, measuring 8 mm, was found in the sigmoid colon. A polypectomy was performed, and a low-grade tubular adenoma was also identified in the sigmoid colon. In addition, chronic active ulcerative colitis was observed.

**Figure 3:**
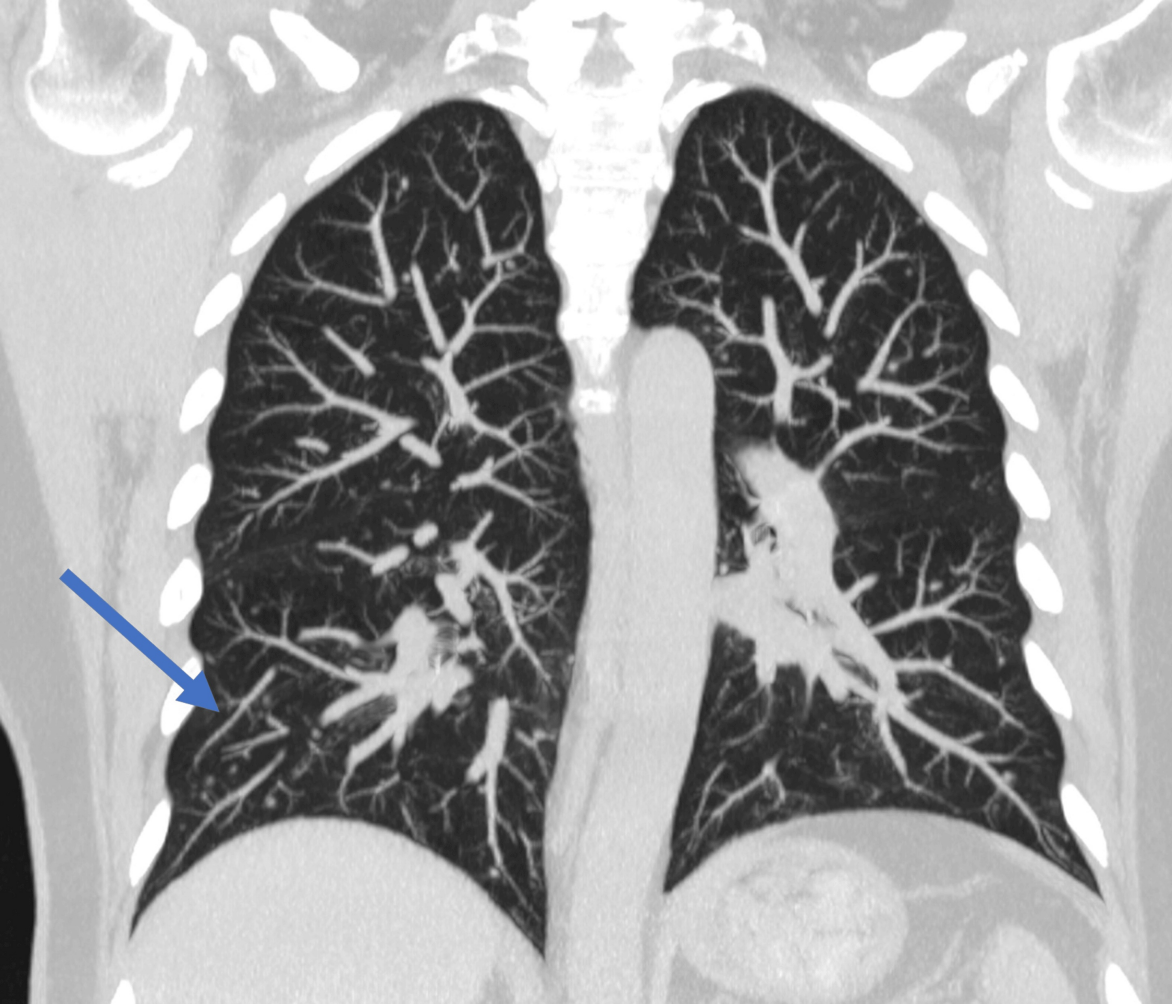
Chest CT coronal plane MIP (maximum intensity projection) performed eight weeks after symptoms shows a significant reduction in bilateral solid nodules (blue arrow).

In the following days, her progress was favorable and she was discharged. A new chest CT scan was recommended two months after the initial symptoms to assess her evolution; the CT scan showed a significant decrease in bilateral solid nodules (Figure 3).

## Discussion

Mpox is a self-limited disease that typically presents with skin involvement. In certain cases, especially in immunosuppressed patients, it can be complicated by gastrointestinal or pulmonary involvement, as seen in the case of our patient.

According to data from the WHO, as of September 30, 2024, 109,699 laboratory-confirmed cases have been reported, including 236 deaths. Among the 10 most affected countries are the United States, Brazil, Spain, the Democratic Republic of the Congo, France, Colombia, Mexico, the United Kingdom, Peru, and Germany, which together account for 79.2% of reported cases worldwide [7].

Mpox is diagnosed through PCR testing of lesion and/or secretion samples. To date, imaging has not been widely used in the diagnosis, as most forms of presentation involve vesiculopustular lesions, and there are no specific radiological findings associated with this disease.

Currently, imaging findings of this disease are limited to case reports and observational studies that describe radiological findings in affected patients. These findings include the development of proctitis with thickening of the rectal wall and striation of the perirectal fat, associated with lymphadenopathy, abscesses, and other pathology [4].

Pulmonary involvement with the presence of bilateral pulmonary nodules remains poorly understood; only one case has been reported in the literature involving a patient with human immunodeficiency virus (HIV), who developed pulmonary nodules as a result of Mpox infection [8,9]. In our patient, the development of pulmonary nodules was attributed to the virus, as resolution was confirmed by a follow-up CT study two months later. Similarly, the diffuse abdominal pain presented by this patient suggests an unusual presentation of Mpox, without completely ruling out the possibility of ulcerative colitis as indicated by the colonoscopy findings. However, in cases of ulcerative colitis, patients typically present with intense abdominal pain accompanied by blood or mucus in the stool, as well as tenesmus, symptoms that the patient did not report during her hospital stay.

In a patient presenting with skin lesions, other differential diagnoses should be suggested in addition to monkeypox, such as herpes zoster and chickenpox. It is important to highlight that in the case of herpes zoster, lesions can appear in immunocompromised patients at different stages of evolution, with distribution along a dermatome. In our patient, however, the involvement was diffuse. As for chickenpox, it is an exanthematic disease that, unlike monkeypox, is more common in children and has a more centripetal distribution, meaning it is more localized to the trunk. It progresses more rapidly, and it is quite rare for the palms of the hands and soles of the feet to be affected and it generally does not present with lymphadenopathy.

## Conclusions

This case demonstrates that Mpox should be considered among the differential diagnoses in patients presenting with a skin rash of undetermined etiology who have recently traveled. Furthermore, because this virus has not been previously documented in many countries, there is a lack of clinical and diagnostic experience regarding its presentation, radiological findings, and therapeutic and preventive approaches.

Although clinical manifestations are usually mild to moderate, individuals with comorbidities, especially immunosuppressed patients, may develop severe symptoms and complications that can affect organs or even threaten life.
